# Hospitals

**Published:** 1876-09

**Authors:** 


### Hospitals.

### COOK COUNTY HOSPITAL, SURGICAL DEPARTMENT.

Service of Dr. Freer.

May 23. Dr. Freer presented and made remarks on the following cases:

1. *Fracture of Clavicle; outer third.*

Dr. F. remarked that he should apply a dressing devised some years ago by himself. The method consists of the proper application of two strips of adhesive plaster. The first piece, three and a half to four inches wide, is a sling, and serves to raise the arm. It passes beneath the elbow, and laps over the opposite shoulder to a point below the nipple and from the same level behind. The second strip, four inches wide, is an axillary band. It is wrapped at one end firmly on the upper part of the arm, then carried across the back beneath the axilla of the opposite side and returned in front to near the point of starting. This fixes the shoulder back. A wedge may be put beneath the axilla or not; but when the case is one of the outer third it is hardly necessary. It is well to measure with a roller bandage before cutting the strips. The warmth of the body will be sufficient to attach every portion except the ends; until they are warm and adherent, a roller had better be applied over the whole. This is one out of perhaps fifty different ways of dressing fractures of the clavicle. It is sometimes put down as Dr. Sayre's method ; but a diagram and a report of cases in the records of the Illinois State Medical Society for 1858, show that it was described here four years earlier than the date of Dr. Sayre's invention, which was merely a repetition.

2. *Elephantiasis.*

A middle aged man of apparently good constitution, presented this condition in the right leg. The leg and foot appeared like a cylinder. Dr. F. remarked that the pathology must be understood before rational treatment could be had.

These appearances did not imply inflammation, but rather a hypertrophied condition of the skin and connective tissue beneath. In consequence of increased vascular supply, there is a condition of hypernutrition with actual increase of tissue, as a result. Dr. Carrochan maintains that enlargement of the arteries of the part attends the affection, and upon this is based his treatment by ligature of the main artery of the limb. In fact, ligature is the leading treatment of the day, and is supposed to have the effect of diminishing the exalted nutritive action in the part, and thus serves to reduce the hypertrophy. You may observe that the skin has been affected with a chronic eczema and is being treated with "tar ointment" with apparent benefit. The results of ligation thus far have not been encouraging, the affection generally returning in a short time. Dr. Carrochan claims one cure, which was the first operation of the kind on record. Bryant also asserts a cure in a case described in his work on Surgery. Treatment by elastic bandaging has its advocates and is worthy of trial.

3. *Amputation of Foot for Frostbite.*

A young man with a thrifty eruption of acne on his face. The frostbite occurred more than a year ago. Spontaneous separation of the toes took place at the metatarso-phalangeal articulation. He says the toes dropped off in three days as clean as it is now. This is almost incredible, for tendons do not separate so readily. Together with ligaments and connective tissue, they are the last to give way. However there has never been any interference, and we have now an open sore and granulations. So much nature can do. She would have done a little better here if she had had longer flaps. It will be necessary now to excise the ends of the bones. It may be that a better surgical display could be made by going back to the point of Hey's or Chopart's operation. But the good surgeon never does any thing from such a motive. It is a rule in surgery, applying to all amputations, to save all you can. The matter was not so much insisted on ten years ago. We will expose the bones, and first see where we can gain sound bone and sufficient skin to form a flap, rather avoiding the articulations. The tissues were dissected up and the metatarsal bones divided with a saw.

1. *Fracture of Right Clavicle.*

May 26. A strong laboring man received a direct stroke. To-day the dressing will be by Fox's apparatus, consisting of a sleeve of muslin and a padded ring for the opposite shoulder. The wedge for the axilla on the affected side, which Fox recommended, is of but little use. For ease and simplicity this dressing has no superior. Adhesive strips do not serve a very good purpose in hot weather for the perspiration throws them off.

2. *Re-amputation of the Leg; lower third.*

A young man, pale and rather unhealthy in appearance. The leg was amputated a year ago last July. It was reamputated the December following. It has since been treated with a great deal of care. The flaps, failing to heal over, and appearing tight and bound down, were at one time dissected up and drawn over the liberated end of the bone again. But still it refused to heal. We now have an ulcer; the bone projects a little. A granulating surface, not thicker than a thumb nail, covers it. If we go back to healthy bone and healthy skin, we may fix it so it will heal. Long flaps often turn out as this has done. They always retract sufficiently and are never too long. The amputation was swiftly performed, about two inches of bone being removed, together with the old cicatritial tissue. Antero-posterior flaps were made. Dr. F. remarked that sutures were of doubtful utility and sure to be a source of irritation. Generally, after amputations, there is more or less oozing of blood with inflammatory exudation, which, if the flaps have been closed by suture, causes tension sufficient at times to arrest the circulation and lead to gangrene and sloughing. Even without sutures, it is remarkable how the flaps will adapt themselves and bring their edges into apposition. Of course where you expect union by first intention, the edges must be adapted at first, either by sutures or adhesive plaster. But in hospital practice we hardly expect healing by first intention. In fact open treatment of wounds is rapidly gaining ground, especially in Germany. The worst enemy the surgeon has to encounter is putrid and putrefying animal matter. This danger is so great that it has led to exaggerated endeavors, and we fear sometimes ill-directed. Some surgeons enter the operating room behind an advance guard of carbolic acid spray, in the vain hope of destroying the invisible spores and germs that lie in wait for their victim. Dr. F. continued by saying that he did not very much believe in the doctrine of the destructive influence of microscopic living things. It is not these germs we are after, but the result of putrefaction. A solution of organic matter is a study from first to last. But there will be no living forms until there is putrefaction. If we follow it up from the day when putrefaction begins, we shall discover coming one after another different forms of life, the evolution of species out of the ruins of others; when last changes have occurred the specimen will be pure of putrid matter. It is a great mistake to think of fighting living spores or living germs. We have to fight putrid organic matter, and this must be done by thorough cleanliness, and by efficient and constant irrigation of the part with proper antiseptic fluids. There must not be recesses and hiding places in which the enemy may lurk, but every part must be accessible to the means of ablution.

May 30. Dr. F. remarked of the leg amputated at the last clinic that the patient had an attack of septicæmia. He had three strong chills in quick succession, followed by profuse perspiration. He had been in a very bad state yesterday; but he had had the same symptoms after the previous operation; so it was to be hoped he would yet do well. There is a difference between septicæmia and pyæmia. The first is the contamination of the system with fluids in a state of putrefaction. A system sufficiently vigorous may cast off the poison, but if the case goes on to pyæmia we have a graver state of things. Local inflammation takes place, as in the lungs, liver, etc., leading to purulent collections, with grave constitutional disturbance, usually ending in death.

1. *Carious Astragalus. Removal.*

A healthy looking young man, eighteen; fireman to an engine. Considerable swelling surrounding the outer malle olus; a fistulous opening just beneath another posteriorly. He says the instep gave him pain last September — the first notice that anything was wrong. In a month and a half the swelling had developed into an abscess, which was opened. He is unable to assign any cause, but it is probable there was some injury to the joint or its appendages; possibly even the bones of the articulation. These bones are quite vascular, and ever so small a rupture of a blood vessel may lead to inflammation which may go on, and at last get to a serious result. The difference between inflammation of bone and inflammation of soft tissues is principally one of time and degree of destruction of tissues that follows. Caries of the tarsus is a very complicated affection. The ankle joint proper is less frequently affected than other portions below. The trouble here is probably between the astragalus and the os calcis. We will proceed to give this foot a trial for its life. It shall have the benefit of a doubt. The attempt will be made in every way to save it in some shape. Dr. F. made a crescent shaped incision, commencing in front of the malleolus, deeply dividing the tissues to the inferior surface of the astragalus. He remarked of Esmarck's band that it was of incalculable value in surgery, preventing any interference from blood, securing a perfectly dry wound. The astragalus was soft and carious, and was scraped out easily with a bone gouge. The whole was involved, and the whole had to be removed. It was the work of about twenty minutes. Communication through the walls left between the cavity and the inner surface of the instep was made and a pledget of oakum drawn in to secure perfect drainage. A plaster of Paris splint was applied to the anterior surface of the leg, instep, and dorsum of the foot, afterward a fracture box.

June 2. The foot operated on for caries at the last clinic is doing splendidly. There is less pain than before, and we may hope for a good result. The cavity will gradually be filled up.

1. *Railroad Injury of Foot.*

A lad attempting to steal a ride on a freight train got his big toe crushed off and considerable contusion to the foot. It has been left that we might see how much could be saved. The first and second phalanges have dropped spontaneously, leaving a large open ulcer from the inner surface to the middle of the dorsum. The operation consisted in disarticulating the whole of the first metatarsal bone. Dr. F. repeated that it was a cardinal principle now-a-days to make the least sacrifice possible. It has become an axiom in surgery that the ratio of accidents increases as operations go toward the body. There is more danger in the ankle than in the foot; more in the leg just above than in either. Always taking into consideration the safety of the patient and a good result, it is sometimes a nice question, therefore, to determine how much to take. This patient is young and vigorous; the growth of tissue is going on very rapidly. It will be left to heal by open granulation. Dressing of oakum.

2. *False Anchylosis of Knee.*

(April 14. Dr. Bogue.) The patella is free, but the position is about the same as before any thing was done. Drs. B. and F. have been studying this case a long time, and are very anxious to straighten it. A full board of surgeons to-day decided to bore the femur and afterward break it. The drill entered about the middle of the lower third. A dozen perforations of the bone were effected through three punctures of the skin. When the softening which results from the perforation is sufficient, the bone will be broken and the limb straightened enough to have the improvement desired.

1. *Injury of Elbow.*

June 6. Child from the dispensary. We should never fail to ascertain whether the joint is capable of all its motions, and never be afraid of hurting the patient. If there is much pain use chloroform. Familiarity with the joints should be acquired above all things. There is nothing in this case but a sprain. Even here we are liable to false anchylosis from inflammation. The advice of Hamilton is good. We should move the joint in a week whenever there may be danger of anchylosis ; flex it, no matter what the injury. Passive motion may save a suit for malpractice.

2. *Injury of Elbow.*

(May 9. Dr. Powell; April 26, Dr. Bogue.) Youth. Here there was a fracture of the external condyle. The head of the radius was removed from its natural position. The hand cannot be carried to the mouth. Passive motion continued.

3. *Dislocation of Sternal End of Right Clavicle, Forward.*

A man past middle age. Two weeks ago he fell down stairs all in a heap. Dr. F. remarked that this injury was very rare. This was the first case he had ever seen. There is some hard swelling, as if there had been a fracture and callus had been thrown out. Whenever he moves the shoulders back the free end is thrown into position and the irritation produced by every movement doubtless causes the indurated swelling. He is to be kept on his back. No other treatment is called for.

4. *Chronic Rheumatic Arthritis of Knee.*

An elderly man. Dr. F. remarked that joints are sparsely supplied with blood vessels. The tissues entering into their formation are of a low order, and are less capable of resisting the vicissitudes of temperature and other insults than those with higher endowments of vitality and nutrition. In this case we have the results of protracted rheumatic disease. The joint is not anchylosed but is stiff. It moves dryly and with creaking sounds. There is little proper secretion from the synovial membrane. Here on the inner side we have a calcarious deposit. The affection has lasted years and we cannot expect a cure. The most rational thing to do is to place the part in a state of immobility and protect it from the vicissitudes of temperature with cotton wadding or fur. The joints of old persons once in a pathological state, require constant protection. Warmth is of the first importance to young and old. Rubbing is of great importance, for it accelerates the circulation and consequently nutrition. There are more salutary effects from *massage*, as the French call it, than the medical profession are aware of. The treatment ordered was a plaster of Paris splint lined with cotton batting, and to go about with a cane.

5. *Chronic Inflammation of Knee.*

A well formed and well nourished man, thirty-three, a cripple for the last three years. The knee is greatly enlarged. There is a sinus beneath. This case illustrates a large class. The hospital is always stocked with them. It is very important here to know whether the inflammation at first was of the synovial membrane, or outside the joint. If it had been in the bones primarily, there would have been destruction before this. Besides bone disease is more an affection of youth. If it had commenced outside, it should have been well long ago. It was doubtless of the synovial membrane, and went on without any great destruction; a chronic subacute case falling short of suppuration, passing the bounds of normal nutrition only a very little. There is probably a granulated or fungous state of the synovial membrane, described as gelatinous. Brodie first described the affection in these terms. It is merely descriptive of the appearance of the tissues within the joint in chronic inflammations of the knee. There is very little anchylosis, and yet he is unable to walk or to use the leg. We are giving the joint a trial, bringing all the evidence to bear upon it, and if at last there should be any doubt the joint ought to have the benefit. We propose now to try the effect of extension for a little while, then we shall put on a plaster of Paris splint. Added to this we shall inject carbolic acid, two per cent. Prof. Haeter of Germany, is the author of this treatment.

6. *Varicose Veins.*

A young carriage maker. The right internal saphenous vein is at fault. The blood is not supported in little segments between the valves, as in health, but in a long column. Dr. F. remarked that after many years experience he prefers the treatment by pins and ligatures to any other method. We want to try to fix the vein between the thumb and finger. At first a vertical insertion of the pin is required; then a sudden pass beneath the vein. Small pins are best, but not too small. The ligature should be drawn sufficiently tight to strangulate the vein. That is all that is needed. The injection of the tincture of chloride of iron has caused disaster in two cases in this hospital, and for that reason is disliked. Any tyro can insert the pins. The time to remove will depend on the effect. If the blood in the segments of the vein included is reduced to a hard coagulum, the time has sufficed. It will be five to seven days. He should be kept in bed; fomentation may be necessary, but the degree of inflammation will depend on the case. Some have such tissues that they resent any thing.

1. *Ulcer of Leg, Following a Burn.*

June 9. Dr. F. remarked that the history of ulcers, properly understood, is the key to that of most injuries of the body. Their study affords more knowledge as to the processes of healing than can be acquired in any other way. In this case the surface of the ulcer has a watery or a gelatinous character. This is not healthy. As soon as the granulations become firm we shall try what virtue there is in grafting.

Dr. F. showed the method he has devised for irrigating wounds, and thus preventing putrid absorption, illustrating it by the case of excision of the astragalus, (May 30.) The leg is suspended in a sling supported by a frame-work above the member, on which is a basin containing a solution of alcohol. In this rests the end of a quarter inch rubber tube, which leads downward to the ankle, and through the wound to the opposite side. The part in the wound is perforated here and there, in order to secure perfect irrigation. The result is a constant dripping into another basin situated beneath. The wound seems perfectly clean, and there is not the slightest evidence of inflammation in the part, nor the least odor.

### Medical Journal and Examiner.

EDITOR: WILLIAM H. BYFORD, A. M. M. D.,

ASSOCIATE EDITORS: JAMES H. ETHERIDGE, M. D.; NORMAN BRIDGE, M. D.; JAS. NEVINS HYDE, A. M., M. D.; FERD C. HOTZ, M. D.

*Published under the auspices of the Chicago Medical Press Association.*

ISSUED TWELVE TIMES A YEAR.

☞ **TERMS, FOUR DOLLARS PER ANNUM, IN ADVANCE; POSTAGE FREE.**

*Address* ~~W. B. KEEN, COOKE & CO~~, Publishers,

113 & 115 STATE STREET.

